# A High-Fat Diet Causes Impairment in Hippocampal Memory and Sex-Dependent Alterations in Peripheral Metabolism

**DOI:** 10.1155/2016/7385314

**Published:** 2015-12-24

**Authors:** Erica L. Underwood, Lucien T. Thompson

**Affiliations:** Cognition & Neuroscience Program, School of Behavioral & Brain Sciences, University of Texas at Dallas, 800 W. Campbell Road, Richardson, TX 75080, USA

## Abstract

While high-fat diets are associated with rising incidence of obesity/type-2 diabetes and can induce metabolic and cognitive deficits, sex-dependent comparisons are rarely systematically made. Effects of exclusive consumption of a high-fat diet (HFD) on systemic metabolism and on behavioral measures of hippocampal-dependent memory were compared in young male and female LE rats. Littermates were fed from weaning either a HFD or a control diet (CD) for 12 wk prior to testing. Sex-different effects of the HFD were observed in classic metabolic signs associated with type-2 diabetes. Males fed the HFD became obese, and had elevated fasted blood glucose levels, elevated corticosterone, and impaired glucose-tolerance, while females on the HFD exhibited only elevated corticosterone. Regardless of peripheral metabolism alteration, rats of both sexes fed the HFD were equally impaired in a spatial object recognition memory task associated with impaired hippocampal function. While the metabolic changes reported here have been characterized previously in males, the set of diet-induced effects observed here in females are novel. Impaired memory can have significant cognitive consequences, over the short-term and over the lifespan. A significant need exists for comparative research into sex-dependent differences underlying obesity and metabolic syndromes relating systemic, cognitive, and neural plasticity mechanisms.

## 1. Introduction

The global prevalence of high-fat diets has led to an epidemic of obesity, insulin-resistance, and type 2 diabetes. Only one-third of American adults are of “normal” weight. One-third of children presenting with diabetes are diagnosed as type 2 [1], a clinical diagnosis formerly rare in children, and often attributed to obesity-induced insulin-resistance. Systematic studies assessing a comprehensive range of systemic metabolic, cognitive, and neuronal deficits linked to diet-induced glucose dysregulation are rare, while studies assessing these domains in reproductively normal females are nearly nonexistent. In the US, the NIH has recently mandated the inclusion of both males and females in clinical research, directing attention to the importance of sexual dimorphism in disorders where sex as a variable has been largely ignored.

Dietary induced obesity has previously been shown to impair performance in a spontaneous alternation task, a measure of hippocampal-dependent spatial memory, while administration of intrahippocampal insulin improved performance [2]. It has been well documented that rats with hippocampal lesions are impaired in a variety of spatial learning tasks requiring integration and use of environmental cues [3–9]. One such task is spatial object recognition, where successful memory is assayed by the relative amount of time that a subject spends with a familiar object moved to a novel location during testing, that is, recognition of the object's change in spatial location between trials. Rodents with hippocampal damage are unable to successfully recognize the moved object [10].

Animals fed high-fat diets show significant potentially pathological changes in hippocampus, including reduced dendritic spines in CA1 [11] and impaired LTP [12] along with memory impairment. Comprehensive comparisons of diet-induced systemic dysregulation of glucose control and of concordant impairment of cognitive function, that is, in both males and females, are needed but unfortunately rare.

The experiments presented here address major sex-dependent diet-induced alterations in systemic metabolism, along with sex-independent severe cognitive (memory) deficits, of rats fed a high-fat diet (HFD) compared to littermates fed a control diet (CD) from weaning.

## 2. Materials and Methods

### 2.1. Subjects

Young adult littermate Long-Evans (LE) outbred rats were socially housed on a 12 hr light/dark cycle with* ad libitum* access to food and water according to their assigned diet, with different diet cohorts housed in different cages from weaning (3 wk of age). Daily records of weight were maintained throughout the study. All procedures were conducted with approval of the Institutional Animal Care and Use Committee of the University of Texas at Dallas in accordance with the guidelines of the USDA.

### 2.2. Diet

All subjects were fed from weaning their assigned diet for 12–15 wk prior to physiological and behavioral assessment. Control diet (CD) groups received 14% fat, 64.8% carbohydrate, and 21.2% protein rat chow (Open Source Diets) along with pure filtered water. High-fat diet (HFD) groups received 58% fat, 25.5% carbohydrate, and 16.4% protein rat chow (Open Source Diets chow augmented with saturated fat (coconut oil) and casein protein) to induce metabolic changes. Nutritional sufficiency of the modified data was assayed and confirmed by Open Source Diets.

### 2.3. Fasting Blood Glucose

Prior to testing, subjects were fasted overnight (8–10 h) to deplete glycogen stores and reduce baseline variability between subjects. Blood samples were obtained by tail nick from well-handled behaviorally naive rats in cohorts of 4 to 8 males and females. Subjects were handled for 1 h prior to testing to reduce stress related fluctuations in blood glucose. Blood glucose levels (mg/dL) were assessed with an AlphaTRAK whole-blood glucose monitor (Abbott Laboratories) and AlphaTRAK 2 test strips. Calibration of the glucose meter was confirmed weekly using AlphaTRAK 2 control solution.

### 2.4. Oral Glucose-Tolerance Testing (GTT)

Glucose-tolerance testing was performed to assess a primary symptom of type 2 diabetes. Again, prior to testing, subjects were fasted overnight (8–10 h) to deplete glycogen stores and reduce baseline variability between subjects. Subjects were handled for 1 h prior to testing to reduce stress-related fluctuations in blood glucose. Basal blood glucose levels (mg/dL) were obtained via tail nick with an AlphaTRAK whole-blood glucose monitor (Abbott Laboratories). Subjects then immediately received either an oral bolus of glucose (2 g/kg) or 0.9% of saline via intragastric lavage tube, and blood glucose was assessed every 15 min for 120 min (in well-handled rats, delivery of saline produced no significantly different fluctuation in serum glucose comparing between oral and i.p. methods of infusion (*p* = 0.9), so responses to saline in the GTT and ITT experiments were combined within (but not between) each of the four groups tested).

### 2.5. Insulin-Tolerance Testing (ITT)

Insulin-tolerance testing was performed using the same protocol described for GTT; however, subjects received either an intraperitoneal (i.p.) bolus injection of insulin (1 U/kg) or 0.9% of saline after baseline readings were obtained (in well-handled rats, delivery of saline produced no significantly different fluctuation in serum glucose comparing between oral and i.p. methods of infusion (*p* = 0.9), so responses to saline in the GTT and ITT experiments were combined within (but not between) each of the four groups tested).

### 2.6. Plasma Corticosterone, Leptin, and Estradiol Analyses

Plasma samples were aliquoted into 500 *μ*L tubes and frozen until use to avoid repeated freeze/thaw cycles. The plasma was thawed at room temperature for 1 hr and then diluted appropriately for ELISA assays. Corticosterone (CORT) was assessed via corticosterone (CSCI) ELISA kits (Abcam), leptin was assessed via rat leptin ELISA kits (Crystal Chem), and estradiol was assessed via mouse/rat estradiol ELISA kits (Calbiotech), using an ELx800 plate reader (BioTek) with Gen5 software.

### 2.7. Spatial Object Recognition (SOR) Testing

All behavioral experimentation was carried out in a white plywood box 60 × 60 × 60 cm with 15 × 15 cm gridlines on the bottom (see Figure 6(a)). Objects were identical solid, nonporous, 5 cm aluminum cubes. Large visual cues were located on adjacent walls of the apparatus for spatial orientation. To minimize odor cues, the apparatus and objects were cleaned first with 30% ethanol and then with Micro90 enzymatic cleaner (International Products Corp.) both before testing began and between all trials. The apparatus was in a room without windows, and dim diffuse overhead lighting was used to avoid shadows.

The following protocol was used to assess SOR (see Figures 6(b) and 6(c)). Rats were handled for 7–10 d prior to 3 d of habituation to the apparatus. On testing day, rats were placed into the apparatus with 2 objects in 2 different locations and allowed to explore for 5 min. After a 30 min intertrial interval (ITI), rats were returned to the apparatus for testing and allowed to explore a duplicate set of objects, with 1 moved to a novel location and 1 placed in a familiar location (identical locale to one of the original objects). Behavior was observed remotely via video recordings, and time spent exploring each object was measured. Behavioral videos were independently scored by two or more assistants blind to the experimental groups to avoid bias, with interrater reliability scores consistently >0.97.

### 2.8. Analyses

One-way ANOVAs with repeated measures (values corrected with Tukey's test) were performed using Prism 6 (GraphPad). Data are presented graphically as means ± SEM, with individual data scatter included in some graphs for additional clarity. Numbers of subjects tested for each measure are also shown in each graph.

## 3. Results and Discussion

A total of 21 young-adult male rats fed the CD and 21 young-adult male rats fed the HFD from weaning along with 20 young-adult female rats fed the CD and 19 young-adult female rats fed the HFD from weaning generated the data presented here. Again, all diet- and sex-dependent effects were assessed in matched littermate cohorts (i.e., both males and females from the same litters were assigned to each treatment condition to reduce variance), with results reported as means ± SEM.

### 3.1. HFD Fed Males, but Not Females, Became Obese

**Figure 1:**
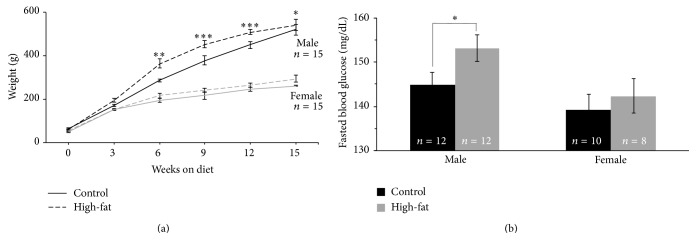
Sex-dependent effects of high-fat diet on body mass and on fasted blood glucose. (a) Males on the HFD gained significantly more weight by week 6 and continued to do so for the remainder of the experiment compared to their CD counterparts. After 15 wk on the diets, control fed males outweighed control fed females by 44%, while HFD fed males outweighed HFD fed females by 50%. Ingesting the HFD did not significantly increase female body weight (^*∗*^ *p* < 0.05; ^*∗∗*^ *p* < 0.01; ^*∗∗∗*^ *p* < 0.001). (b) Male HFD fed rats had significantly elevated fasting blood glucose compared to both control males and HFD females (*p* = 0.05), while no differences were observed between the dietary groups in female rats.

All rats tested gained weight steadily on both diets across the span tested (*F* (15, 108) = 23.31, *p* = 0.0001), with males exhibiting more dramatic weight gains on both diets (see Figure 1(a)). Notably, male rats on the HFD gained significantly more weight than their littermates fed the CD: HFD males had significantly greater body mass than controls on week 6 (*p* = 0.004), week 9 (*p* = 0.0001), week 12 (*p* = 0.0001), and week 15 (*p* = 0.05). However, female rats on the HFD did not gain significantly more weight than their littermates fed the CD. By week 15, males fed the HFD outweighed CD males by 13%, while HFD females outweighed CD females by only 4%. Previous studies suggest that obesity is influenced by sex hormones; female rats gain less weight compared to males when fed a high-fat diet, but this difference is no longer seen after ovariectomy [13]. Our own data (see Figure 4(b)) do not indicate significant alterations in circulating estradiol or in cycle-dependence of subsequent behavioral performance (data not shown) in females fed a HFD. Within a relatively short time on the HFD (3 mo), males became obese while females did not. Since LE rats are an outbred strain, these data may be of greater comparative value than that obtained in studies using inbred strains of rats or mice.

### 3.2. HFD Fed Males, but Not Females, Had Elevated Fasting Blood Glucose

**Figure 2:**
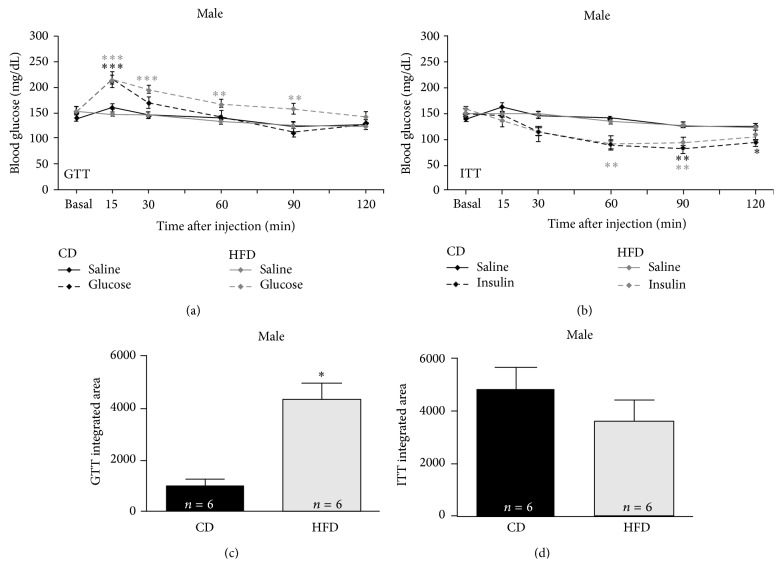
HFD diet leads to failure of GTT in male rats. Glucose-tolerance testing (a) is used clinically as a diagnostic tool for diabetes, while insulin-tolerance testing (b) is informative but not widely used clinically. Comparisons between fasted male rats previously fed the CD or the HFD for 15 wk showed significant differences in blood glucose regulation after an oral bolus of glucose between these diet groups (a). Controls showed a normal rapid rise and fall of blood glucose (significantly elevated only at 15 min after ingesting the glucose bolus), while HFD fed male rats showed significant elevations of blood glucose for 2 hr after glucose bolus. Insulin-tolerance testing showed small diet-dependent differences in male rats, with a faster return to baseline after a bolus injection of insulin in HFD fed males (b), suggestive that at this age HFD males not only remained systemically sensitive to insulin but can successfully utilize exogenous insulin in glucose regulation. Integrated area under the GTT curve (c), but not over the ITT curve (d), was also significantly increased in HFD fed males (^*∗*^ *p* < 0.05; ^*∗∗*^ *p* < 0.01; ^*∗∗∗*^ *p* < 0.001).

**Figure 3:**
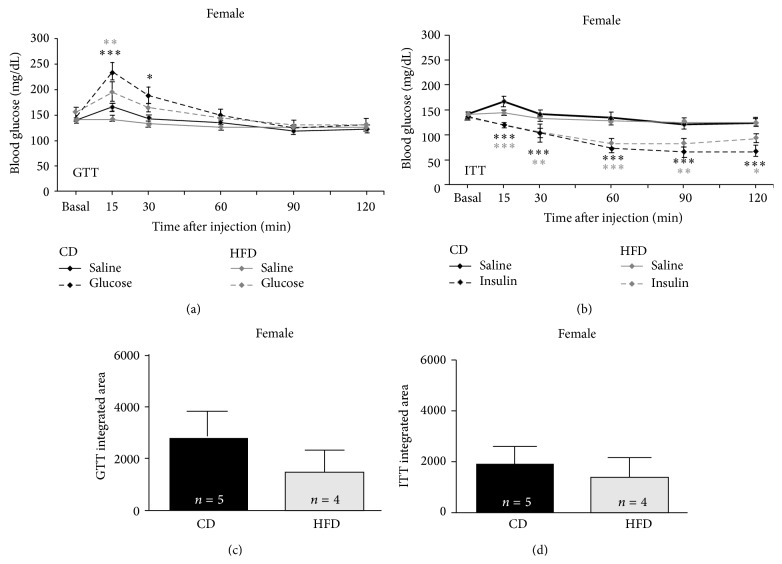
HFD did not significantly alter GTT or ITT in female rats. In contrast to males, female rats on CD and HFD exhibited relatively little diet-dependent changes in glucose- (a) or insulin-tolerance (b). Additionally, no dietary effects were seen in the integrated area under or above the curve (resp.) for either glucose-tolerance testing (c) or insulin-tolerance testing (d).

According to the American Diabetes Association [14], one major criterion for diagnosis of type 2 diabetes in human patients is a significant elevation of fasting blood glucose. Basal fasting blood glucose concentrations (i.e., prior to additional external challenges) sampled from HFD versus CD rats were significantly different in males (see Figure 1(b), *F* (3, 36) = 3.42, *p* = 0.05), with significant elevations in the HFD group compared to controls. However, fasting basal blood glucose was not significantly different in female rats comparing between the two diet groups (*p* = 0.2). Total endogenous circulating glucose has been shown to be greater in men than women due to differences in general body size between sexes [15, 16]. Therefore the body mass (weight) differences discussed previously may contribute to these differences seen in fasted blood glucose levels. As described below (Figures 2 and 3, data from males and females, resp.), when control of circulating blood glucose was challenged, either with a bolus injection of glucose or with a bolus injection of insulin, further sex- and diet-dependent differences were observed.

### 3.3. HFD Impairs Male, but Not Female, Systemic Glucose-Tolerance

Rats on the HFD exhibited sex-dependent alterations in systemic blood glucose responses to a bolus oral infusion of glucose. Compared to physiological saline infusion, blood glucose was significantly elevated in all groups (i.e., males and females, irrespective of diet) tested 15 min after glucose infusion (*F* (16, 112) = 3.758, *p* = 0.001). Sex- and diet-dependent differences in the later sustained magnitude and duration of responses to this glucose challenge (glucose-tolerance) are detailed below.

Blood glucose of male CD rats after an oral bolus of glucose rapidly returned to baseline after its initial rise (see Figure 2(a)) and was not significantly elevated compared to oral saline infused male CD rats at intervals 30 min or more after infusion (*p* = 0.2). Blood glucose of male HFD rats after an oral bolus of glucose failed to rapidly return to baseline (see Figure 2(a)) and was significantly elevated compared to saline infused male HFD rats at intervals 30 (*p* = 0.001), 60 (*p* = 0.01), and 90 (*p* = 0.01) min after injection. Such a sustained elevation of blood glucose would constitute a failed glucose-tolerance test in clinical assays for type 2 diabetes.

Blood glucose of female CD rats after an oral bolus of glucose more slowly returned to baseline after its initial rise (see Figure 3(a)) and was significantly elevated compared to saline infused female CD rats 30 min after the bolus (*p* = 0.05), returning to baseline 60 min or more after infusion (*p* = 0.1). Unlike in males, blood glucose of female HFD rats after an oral bolus of glucose was only slightly less elevated than that of CD female rats infused with glucose 15 min after injection (*p* = 0.08). Blood glucose of female HFD rats after an oral bolus of glucose returned to baseline more rapidly (see Figure 3(a)) than CD females and was not significantly elevated, compared to saline injected female HFD rats, at intervals 30 min or more after infusion. Although responses of females to glucose challenge (glucose-tolerance) were not identical to those of male, HFD females maintained homeostatic control of circulating glucose in a manner similar to that of female controls.

An additional form of analysis, integrated area under the curve (AUC, a measure of total blood glucose elevation across the entire glucose-tolerance test interval, referenced to the equivalent group's saline-infusion curves) was significantly different between groups tested [*F* (3, 16) = 6.048, *p* = 0.006]. No sex-dependent differences in AUC were observed for controls: AUC was not significantly greater for CD females compared to CD males (*p* = 0.3). However, sex-dependent differences in AUC were observed for HFD rats. AUC was significantly greater in HFD males compared to HFD females (*p* = 0.05). Further, AUC was significantly increased in HFD males compared to CD males (*p* = 0.006; Figure 2(c)). This finding further corroborates failure of the clinically relevant glucose-tolerance assay by HFD males detailed above. HFD females did not exhibit an increased AUC compared to CD females (*p* = 0.9; Figure 3(c)), verifying that functional glucose-tolerance was maintained in HFD females. Thus, not only was resting blood glucose elevated, but homeostatic responses to a glucose challenge (glucose-tolerance) were also impaired, only in male but not in female HFD rats.

### 3.4. HFD Slightly Altered Systemic Insulin Sensitivity in Males but Not Females

The HFD also sex dependently altered systemic blood glucose responses to a bolus injection of insulin, but to a much lesser magnitude than glucose-tolerance testing revealed. Compared to physiological saline injection, blood glucose was significantly reduced in all groups tested 60 min after insulin injection (*F* (3, 35) = 5.105, *p* = 0.005) with diet- and sex-dependent variations in the magnitude, onset, and duration of responses detailed below.

Compared to physiological saline injection (see Figure 2(b)), blood glucose of male CD rats injected with insulin was significantly reduced 60 min after injection (*p* = 0.001), still reduced 90 min after injection (*p* = 0.01), and failed to return to baseline even when tested 120 min (*p* = 0.05) after injection. Compared to comparable responses to physiological saline injections of HFD males (see Figure 2(b)), blood glucose of male HFD rats injected with insulin was significantly reduced 60 min (*p* = 0.01) and 90 min (*p* = 0.01) after injection, but returned to baseline within 120 min after injection (*p* = 0.1), that is, faster than controls.

Compared to physiological saline injections (see Figure 3(b)), blood glucose of female CD rats injected with insulin was significantly reduced 15 min after injection (*p* = 0.001), continued to decline 30 (*p* = 0.001), 60 (*p* = 0.001), and 90 min (*p* = 0.001) after injection, and failed to return to baseline even 120 min (*p* = 0.001) after injection. Compared to the responses of HFD females to physiological saline injections (see Figure 3(b)), blood glucose of female HFD rats injected with insulin was also significantly reduced 15 min after injection (*p* = 0.001), remained reduced 30 (*p* = 0.01) and 60 min after injection (*p* = 0.001), and failed to return to baseline when tested 90 (*p* = 0.01) or 120 min (*p* = 0.05) after injection. Insulin more rapidly depleted circulating glucose in female compared to male rats, and recovery of circulating glucose (return to baseline) was slower in females than in males.

Integrated area above the curve (AAC, a measure of total blood glucose reduction across the insulin-tolerance test interval, compared to the equivalent group's saline-injection curves) was not significantly different between groups tested. AAC was not significantly greater in CD females compared to CD males (*p* = 0.1), nor was it significantly greater in HFD females compared to HFD males (*p* = 0.2). There were no significant differences in AAC comparing CD rats to HFD rats, either in male (*p* = 0.6, Figure 2(d)) or female (*p* = 0.9, Figure 3(d)) cohorts. Although ITT is not used clinically in diagnosis of type 2 diabetes, a maintained ability to respond to the hypoglycemia induced by insulin in both HFD and CD rats indicates that the feedback loop integrating the hippocampus to hypothalamus to pituitary to adrenal cortex (HPA axis) remained functionally intact [17] in all groups tested. Systemic changes in glucose regulation in the HFD groups (in particular, the dysregulation exhibited by HFD males) cannot readily be attributed to insulin intolerance in these young rats. Given the classic diabetic signs of elevated basal fasting glucose in the HFD males, it is likely that these male rats responded, like many type 2 diabetics early in their disease progression, by increasing release of insulin in an attempt to regulate their blood glucose. Preliminary data (not shown) from our laboratory supports this hypothesis and makes assessment of systemic insulin-resistance a complex multifactor issue.

### 3.5. HFD Increased Circulating Corticosterone in Males and Females

**Figure 4:**
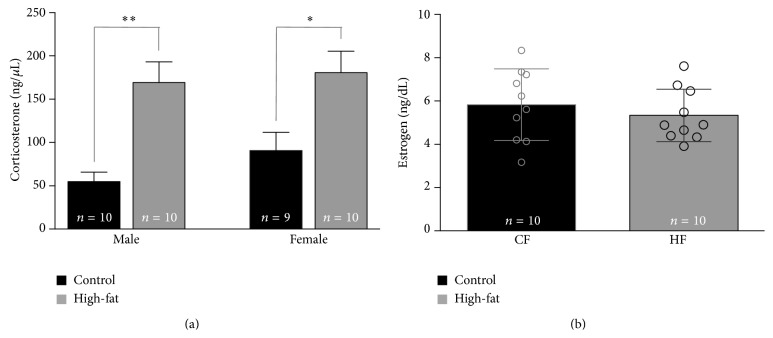
HFD selectively altered circulating corticosterone but not estradiol. The HFD significantly increased circulating corticosterone (a) in both males and females (^*∗*^ *p* < 0.05; ^*∗∗*^ *p* < 0.01; ^*∗∗∗*^ *p* < 0.001). Circulating estradiol levels (b), often asserted to be an experimental confound precluding use of females in physiological and behavioral studies, were not significantly altered in females fed the HFD compared to those receiving the CD.

Resting circulating concentrations of serum corticosterone, a major hormone regulating glucose utilization [18], differed significantly between diet treatments (*F* (3, 34) = 8.15, *p* = 0.0003; see Figure 4(a)), but not between sexes in well-handled rats. Serum corticosterone concentrations were not significantly different in CD fed males compared to females (*p* = 0.6). Corticosterone concentrations were significantly elevated in HFD fed males compared to control fed males (*p* = 0.003) and in HFD fed females compared to control fed females (*p* = 0.03). The high-fat diet did not impact serum corticosterone in males differently from females (*p* = 0.98).

### 3.6. HFD Did Not Influence Circulating Estradiol

Circulating levels of estradiol did not differ significantly between CD and HFD females (*p* = 0.2; Figure 4(b)). Previous studies suggest that obesity is influenced by sex hormones; female rats gain less weight compared to males when fed a high-fat diet, but this difference is no longer seen after females undergo ovariectomy [13]. Compensatory estradiol-mediated mechanisms do not seem to account for the other sex-dependent metabolic differences observed here.

### 3.7. HFD Did Not Elevate Circulating Leptin

**Figure 5:**
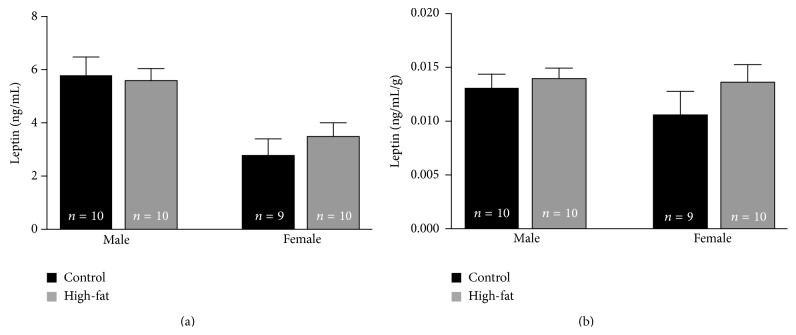
HFD did not significantly alter circulating leptin. Raw data (a) for circulating leptin in males and females appears to show a sex-dependent but not diet-dependent difference in levels of the circulating hormone. However, when leptin concentration is corrected for body weight (b), no significant sex- or diet-dependent differences were observed.

Leptin, a satiety hormone released by adipose cells, was unaffected by the HFD. Although larger males exhibited higher concentrations of circulating leptin (Figure 5(a)), when corrected for body weight [19] no statistically significant sex- or diet-dependent differences in circulating leptin were observed in young LE rats (Figure 5(b)).

### 3.8. HFD Impaired Hippocampal-Dependent Spatial Memory in Both Sexes

**Figure 6:**
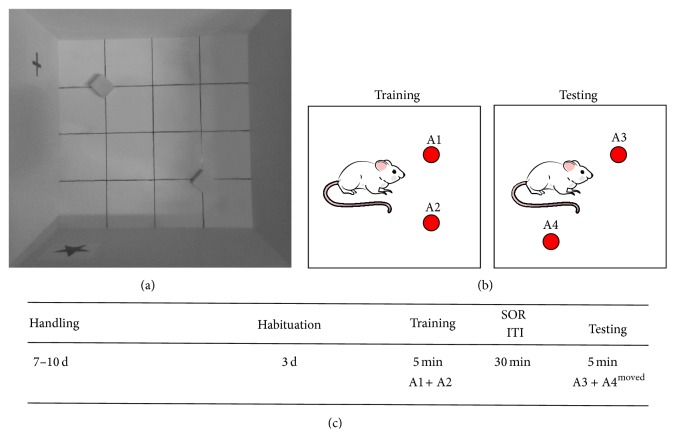
Memory assessed via spatial object recognition (SOR). The task was performed in a 60 × 60 × 60 cm walled open field, with 15 × 15 cm gridlines on the floor and spatial cues positioned on adjacent walls (a). Identical objects were 5 cm solid aluminum cubes. For training, rats were allowed to explore the open field with two objects in defined locations. For testing, rats were again allowed to explore the open field, now with one object in the original location, and one object moved to a novel location (b). The subjects were fully acclimated to handling and to the open field prior to training and testing (c).

**Figure 7:**
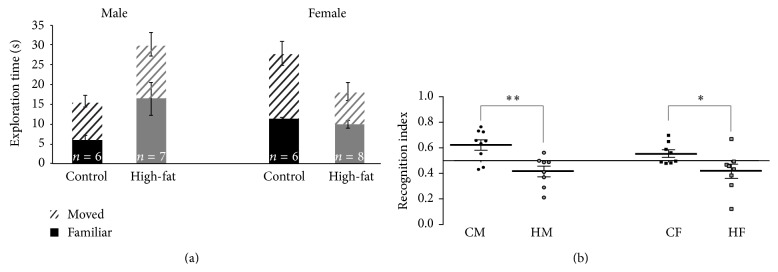
HFD impairs spatial memory independent of sex. No sex- or diet-dependent differences were observed in total object exploration (a), total line crosses, centerline crosses, or time spent in the center (data not shown). However, HFD effects were reported in recognition index (b), the comparison of time exploring object in novel location to total time exploring objects (^*∗*^ *p* < 0.05; ^*∗∗*^ *p* < 0.01; ^*∗∗∗*^ *p* < 0.001).

Spatial memory, assessed via recognition index in a spatial object recognition task (SOR), was significantly impaired in both male and female rats fed the HFD (*F* (3, 28) = 10.24, *p* = 0.0001, Figure 7(b)). The recognition index was defined as the amount of time spent exploring the novel (moved location; Figure 6(b)) object relative to the total time spent exploring both objects [RI = *T*_*N*_/(*T*_*N*_ + *T*_*F*_)] and was used as the primary measure of memory retention [20]. There were no significant differences in total time spent exploring the objects (*p* = 0.7). Additionally, no significant differences were found in other measures of exploration (total line crossings, *p* = 0.2) or of anxiety (center line crossings, *p* = 0.2; time in center, *p* = 0.3), indicating that the memory impairment cannot be attributed to disparities in motor activity or other performance variables between groups (Figure 7(a)). Prior studies have documented HFD-dependent learning and memory impairments in male rats performing hippocampal-dependent tasks [21–23]. McNay et al. [2] found spatial memory impairments in male Sprague-Dawley rats with HFD induced obesity (defined as the top tertile by weight gain within their cohort), but no impairment in HFD-resistant rats (defined as the bottom tertile by weight gain). In our hands, there was relatively little variability in distribution of body weights within our diet groups, for both males and females (note the small variance across different ages in Figure 1(a)). While our findings for obese males are concordant with those of McNay et al. [2], the cognitive deficits shown here in HFD females were not accompanied by obesity, by major systemic metabolic changes indicative of onset of type 2 diabetes, nor by peripheral glucose dysregulation. Indeed, the results of the current study strongly suggest that young females ingesting a high-fat diet may be at high cognitive risk, since they may remain largely asymptomatic on systemic measures likely to be clinically assessed (i.e., measures associated with type 2 diabetes: BMI, fasted blood glucose, and glucose-tolerance testing). Our findings highlight an imperative for more research into sex differences, specifically those relating systemic and neural plasticity mechanisms in metabolic disorders, and should be extended across the lifespan.

## 4. Conclusions

In our rat model, significant weight gain (obesity) was readily induced in male but not in female LE rats ingesting a high-fat diet for approximately 12 weeks (Figure 1(a)) compared to littermate controls. Additionally, diagnostic criteria for type 2 diabetes, including elevated fasting blood glucose (Figure 1(b)) and an impaired glucose-tolerance test (Figures 2(a) and 2(c) [14]), were met by young male but not female HFD rats.

It is important to note that obesity in this and in a large majority of published studies is defined as an overall increase in total body weight [24] compared to controls, in this case induced by obligate consumption of the HFD compared to littermates on standard diet. Systemic measures further validated our model of obesity and metabolic dysregulation in males but not in females, mandating further study to explain these sex-dependent differences. Systematic assessment of peripheral or abdominal body fat is a complex issue [25]. While a previous study of the effects of HFD on cognitive and neural function in middle-aged male Fisher-344 rats found several significant diet-induced increases in different body fat stores and a weak correlation (*r* = 0.3) between measures of systemic lipids and memory measures [24], no significant diet-induced memory impairments were observed. Future work in our and other labs will continue to probe potential lipid-related links.

Our investigation of sex-dependent effects of ingestion of a high-fat diet on young adult rats examined multiple systemic metabolic markers, including these diagnostic of diabetes, and found that despite sex-differences in a variety of these markers, memory performance was equally and significantly impaired on a hippocampal-dependent spatial object recognition task in both male and female rats fed the HFD (Figure 7(b)). Ongoing studies in our laboratory have assessed diet- and sex-dependent changes in hippocampal function which will be reported separately, and diet-induced changes in other brain regions with significant cognitive roles (including neocortex and basolateral amygdala) remain areas of interest. While diet-induced memory impairments have been consistently linked with systemic metabolic impairments when testing has been carried out exclusively in male rodents and other model systems, our divergent findings highlight and reemphasize the need for inclusion of female subjects and direct and systematic comparison with data from males in future studies.

To our knowledge, these are the first findings of sex-dependent dietary changes in systemic glucose regulation, along with sex-independent impairment of hippocampal-mediated cognitive performance. No compensatory changes in estradiol (Figure 4(b)) nor in leptin (Figure 5(b)) were found in comparisons of HFD females to CD females, so additional signals remain to be explored to account for the lack of other systemic changes in glucose regulation in HFD females. Of the systemic variables assessed, corticosterone alone was significantly elevated in both HFD males and females (Figure 4(a)) and could potentially impact memory performance. Male rats subjected to chronic stress with enhanced circulating corticosteroids fail to remember platform location in a spatial Morris water maze task [26]. However, studies assessing chronic stress (and subsequent corticosterone increase) in females report enhancements in spatial memory performance [27]. While elevations in corticosterone could account for memory impairments seen in male HFD rats, it would not explain the impairment seen in HFD females and require further study.

Additional systemic metabolic markers, as well as an extensive range of signaling pathways within the central nervous system, also remain to be addressed. As noted, other studies in our laboratory have actively explored central effects of the HFD on intrinsic excitability, insulin-sensitivity, and glucose- and insulin-signaling pathways in hippocampal CA1 pyramidal (excitatory output) neurons, as well as on performance on other hippocampal-dependent memory tasks, and continue to strengthen the case for the need for comparative studies in both males and females under the same conditions. Since hippocampal neurons are reciprocally connected with numerous neocortical regions and cortical neurons also express abundant IRs (and insulin-resistance has been reported in cortical regions of Alzheimer's patients) [28, 29], the effects of HFD on hippocampus, neocortex, and other brain regions will continue to be assessed in future studies of HFD-related cognitive decline.

While the consequences of sex differences in development and impact of type 2 diabetes can be profound, comparative metabolic studies in young and young adult model systems are rare, despite alarming human population trends in youth [1]. Obese women with type 2 diabetes have a higher occurrence of cognitive decline than men [30] as they age. Obese women are twice as likely to have dementia as women of normal weight, while obese men are at no greater risk than normal weight men [31]. Long-term consequences of obesity, glucose dysregulation, and consequent neuronal dysfunction must be studied in parallel in males and females, since a one-size-fits-all approach cannot adequately detail or identify all relevant issues.
